# Supplementary figures and images for: Repurposing an endogenous degradation domain for antibody-mediated disposal of cell-surface proteins
Source: EMBO Rep. 2024 Jan 29;25(3):8. doi: 10.1038/s44319-024-00063-3 (PMC10933360; doi:10.1038/s44319-024-00063-3)

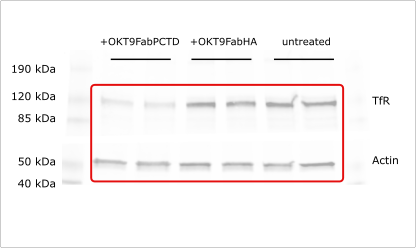

Supplement: Supplementary file 1 — Source Data Fig. 1 [file 44319_2024_63_MOESM1_ESM.zip › SD Figure 1/1E/1E.tiff]

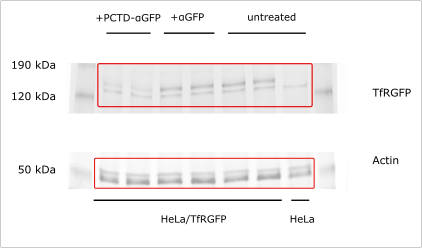

Supplement: Supplementary file 1 — Source Data Fig. 1 [file 44319_2024_63_MOESM1_ESM.zip › SD Figure 1/1C/1C_blots.tiff]

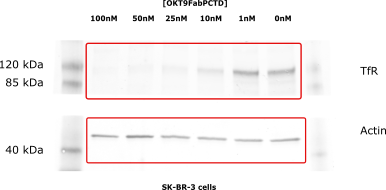

Supplement: Supplementary file 2 — Source Data Fig. 2 [file 44319_2024_63_MOESM2_ESM.zip › SD Figure 2/2A/2A.tiff]

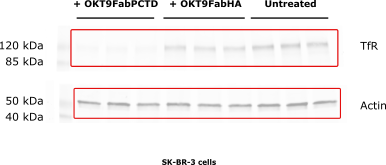

Supplement: Supplementary file 2 — Source Data Fig. 2 [file 44319_2024_63_MOESM2_ESM.zip › SD Figure 2/2C/2C.tiff]
