# Supplementary material for: Repurposing an endogenous degradation domain for antibody-mediated disposal of cell-surface proteins
Source: EMBO Rep. 2024 Jan 29;25(3):8. doi: 10.1038/s44319-024-00063-3 (PMC10933360; doi:10.1038/s44319-024-00063-3)
Supplement: Supplementary file 6 — Expanded View Figures [file 44319_2024_63_MOESM6_ESM.pdf]

Expanded View Figures

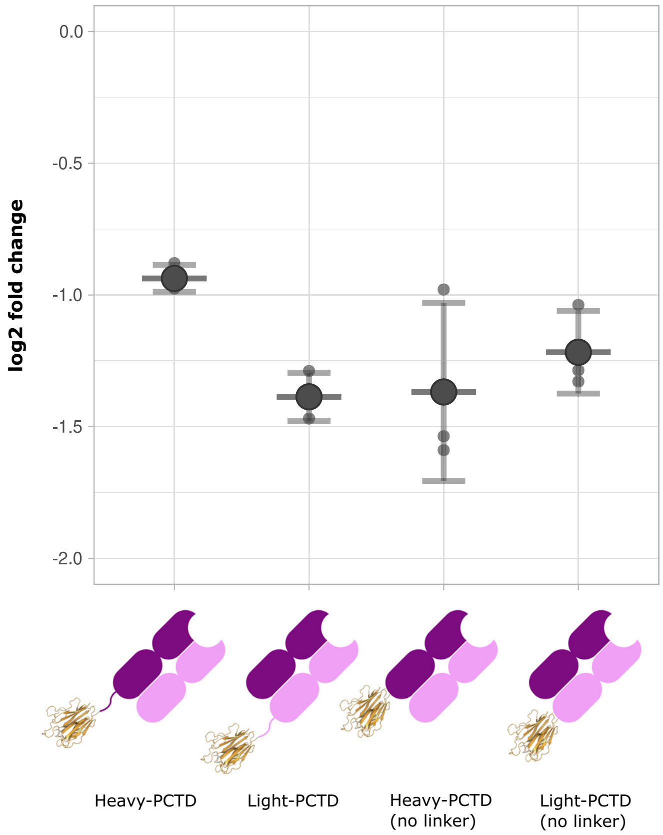

Figure EV1. PCTD fusion format does not significantly affect efficiency of OKT9FabPCTD degradation.

SK-BR-3 cells were treated with 25 nM of four different OKT9FabPCTD fusions and TfR degradation measured by Western Blot and densitometry and shown as log(2)-fold change. The different fusion formats are depicted as cartoons, with OKT9 heavy chains shown in dark purple, light chains shown in light purple, and PCTD shown in gold as a molecular model. 35 amino acid glycine/serine linkers are depicted as solid lines separating OKT9 from PCTD. 3 biological replicates per sample are plotted as separate data points together with mean and s.e.m. One-way ANOVA indicates no significant differences ($p > 0.05$) between the different formats.

© The Author(s)

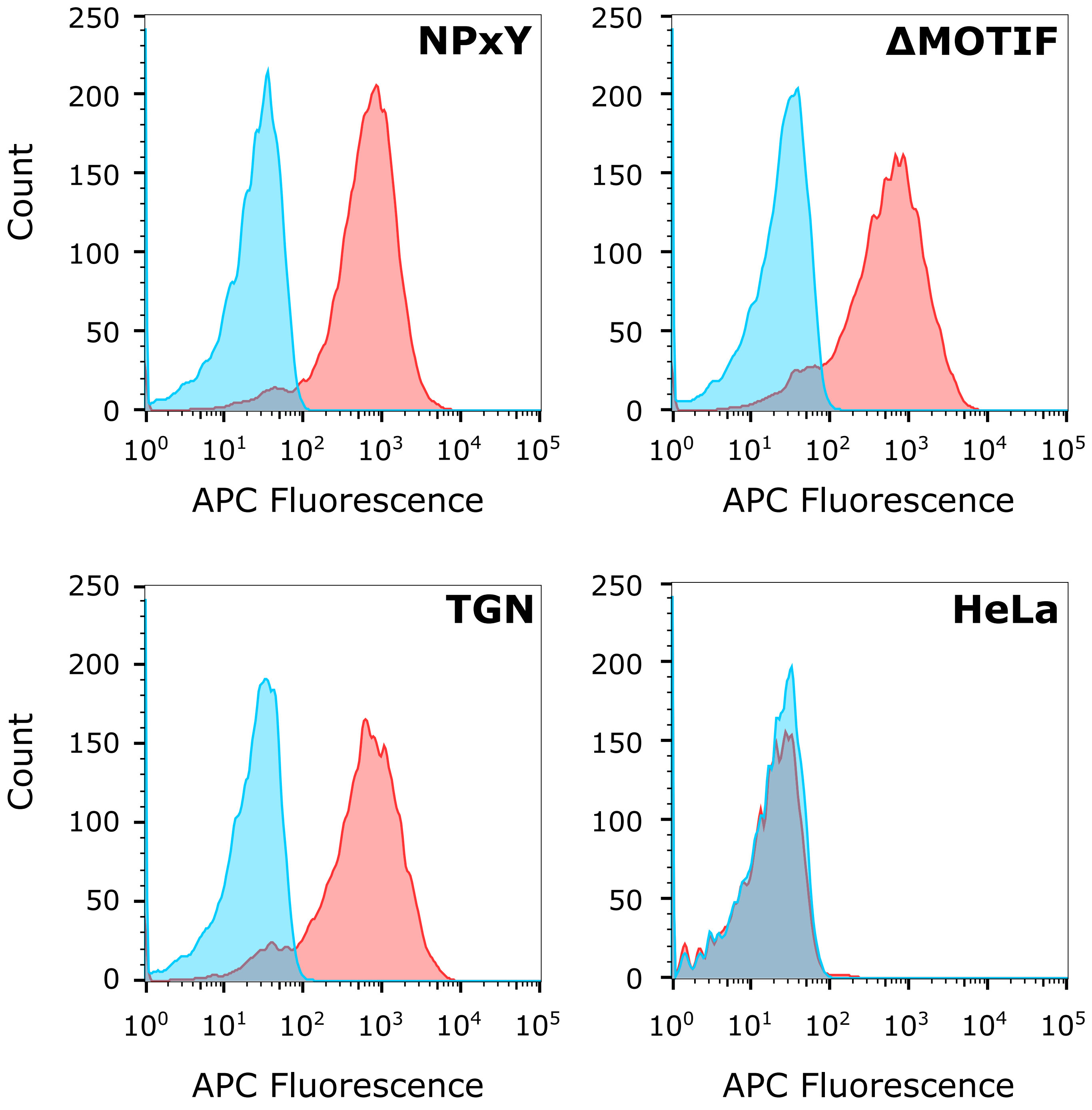

Figure EV2. Reporter cell lines can bind and/or take up fluorescent CD8 antibody.

Reporter cells, or HeLa cells as a negative control, were incubated with an allophycocyanin (APC)-labelled anti-CD8 α chain antibody for 30 min or left untreated, then washed in PBS and harvested for flow cytometric analysis. Because the reporter cell lines are fluorescent due to the presence of GFP, compensation was performed using Becton Dickinson CompBeads. Traces are depicted for a representative sample from two biological replicate experiments; red traces depict cells exposed to anti-CD8 antibody and blue traces depict untreated cells. All reporter cell lines display similar levels of anti-CD8 antibody binding/uptake, whereas HeLa cells do not take up the antibody.

 © The Author(s)

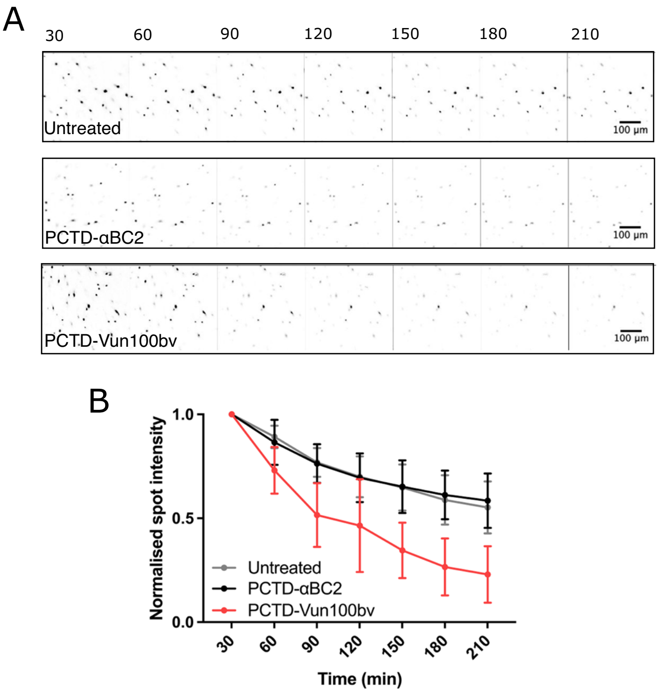

Figure EV3. PCTD-Vun100bv induces US28 degradation in fibroblasts, whereas non-US28-targeting PCTD-αBC2 does not affect US28 levels.

(**A**, **B**) Fibroblasts were infected with HCMV-US28-GFP. Three days post-infection, cells were treated with PCTD-Vun100bv, with a non-US28-targeting PCTD-αBC2 control, or left untreated, and then imaged using live-cell fluorescence microscopy. (**A**) Time course series generated with live-cell fluorescence microscopy. Numbers indicate minutes post-treatment, starting with 30 min post-treatment. Scale bars: 100 µm. (**B**) Quantified normalised change in per-spot intensity of fluorescence signal from (**A**). A two-way ANOVA with Tukey's test shows a highly significant difference from 60–210 min between PCTD-Vun100b and untreated ($P < 0.0001$), but no significant difference between control and untreated. Data is shown as mean ± SD and is representative of at least 5 technical replicates per condition.

© The Author(s)

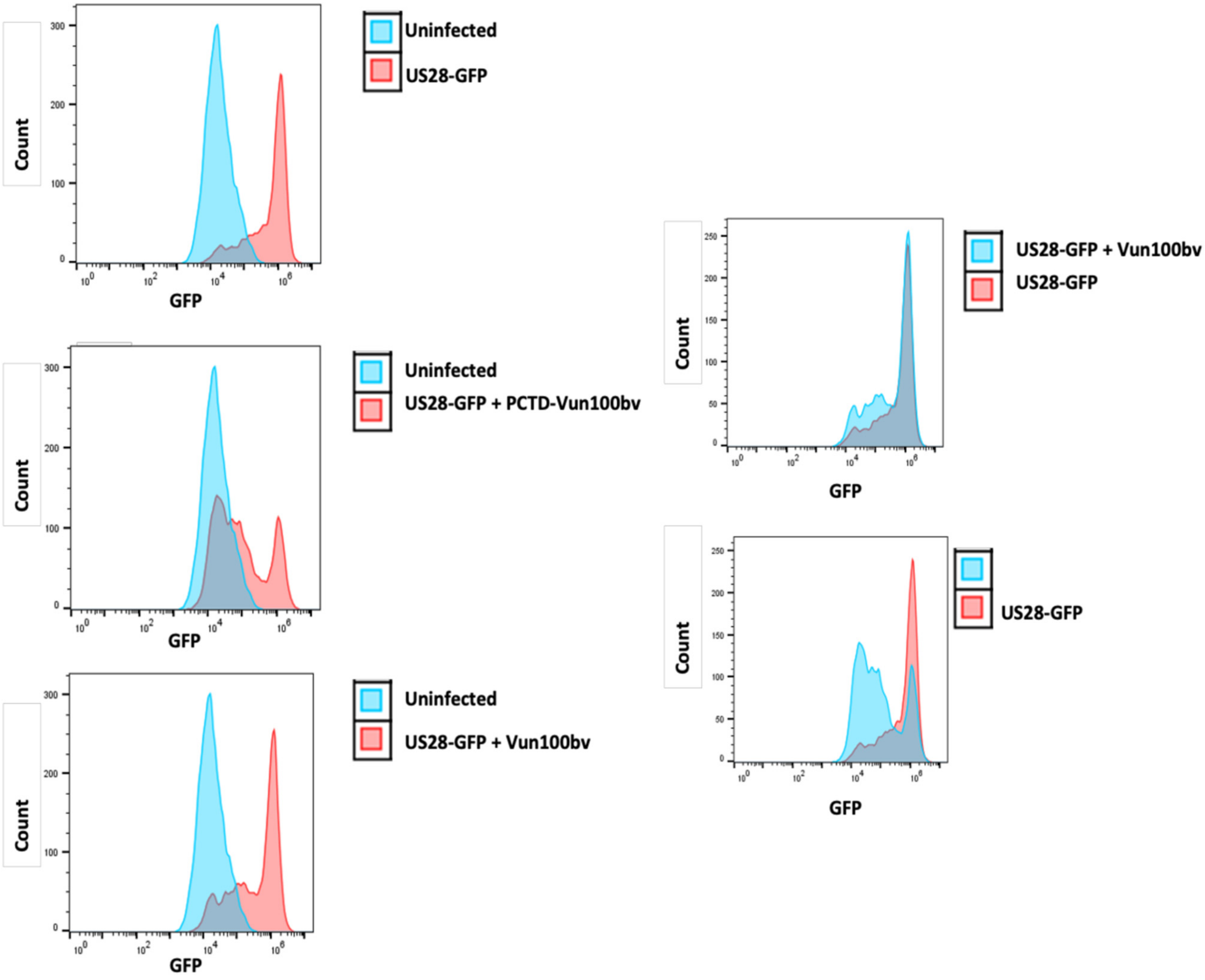

Figure EV4. PCTD-Vun100bv degrades US28 in fibroblasts, whereas Vun100bv treatment does not affect US28 levels.

Fibroblasts were infected with HCMV-US28-GFP and were left untreated or were treated with Vun100bv or PCTD-Vun100bv three days post-infection. Total US28 levels were quantified by measuring fluorescent GFP intensity via flow cytometry. The data is representative of two biological replicates.

 © The Author(s)
